# What influences consumers’ online medication purchase intentions and behavior? A scoping review

**DOI:** 10.3389/fphar.2024.1356059

**Published:** 2024-02-13

**Authors:** Yam B. Limbu, Bruce A. Huhmann

**Affiliations:** ^1^ Department of Marketing, Montclair State University, Montclair, NJ, United States; ^2^ Department of Marketing, Virginia Commonwealth University, Richmond, VA, United States

**Keywords:** online pharmacy, scoping review, over-the-counter (OTC) medication, prescription drug, purchase intention, purchase behavior

## Abstract

**Objective:** Consumers increasingly buy pharmaceuticals online. No scoping review has been carried out to summarize and synthesize the studies that have identified drivers of consumers’ purchase intention and behavior from online pharmacies. Thus, we conducted a scoping review to explore the extent to which prior research has studied consumer purchase intentions and behavior related to online pharmacies, the drivers previously identified to explain consumers’ online pharmacy purchase intentions and behavior, and how these antecedents differ between OTC and prescription medications. Then, we identified gaps in the published literature to form a comprehensive theory-based agenda for future research.

**Methods:** We searched PubMed, Web of Science, and Scopus to retrieve relevant studies published in English in peer-reviewed journals. The search strategy identified forty-eight eligible studies.

**Results:** We identified twelve types of factors influencing purchase intentions and behaviors from online pharmacies: demographics, convenience, availability, price, evaluations of the purchase environment, information sources, internet usage, prior experience, perceived risk, health insurance, privacy, and product. Our analysis also revealed differences between OTC and prescription medications in drivers of purchase intentions and behaviors.

**Conclusion:** While demographic factors tended to be the most often measured influences on intentions and behavior, their role was generally inconsistent, with many contradictory results. However, other factors (e.g., convenience, availability, lower prices, and favorable evaluations toward the purchase environment) more consistently enhanced online medication purchase intentions and behavior. An extensive agenda for future research is advanced.

## 1 Introduction

Consumers increasingly purchase products online; for example, rising from 53% of consumers in the United Kingdom (UK) in 2008 to 87% in 2020 (Office for National Statistics, 2020). Many consumers now prefer to purchase online, including 61.4% in the United States, 57.4% in the UK, 51.6% in Spain, 49.2% in France and Poland, and 47.8% in Australia and Germany (Klarna, 2023). In some countries, many consumers purchase drugs or health products online each year, for example, 43% in Germany, 39% in Mexico, 36% in the UK, 33% in the United States, and 27% in France (Statista Consumer Insights, 2023). Thus, many consumers are turning to online pharmacies to purchase medications.

A survey by the Alliance for Safe Online Pharmacies (2020) found that 35% of American consumers have purchased medication for themselves or someone in their care from an online pharmacy (also known as an e-pharmacy, internet pharmacy, or cyber pharmacy). Online pharmacies sell or dispense pharmaceutical drugs or other government-regulated medical treatments through the internet or electronic media (e.g., websites, social media, or mobile apps). Consumer purchases from online pharmacies continue to grow rapidly due to the convenience, accessibility, and cost savings that they provide (Limbu and Huhmann, 2023b). For example, purchases by Hungarian consumers from online pharmacies increased over tenfold between 2018 and 2020 (Fittler et al., 2022a). Online pharmacies have become a major healthcare resource for consumers, with global sales of US$98.8 billion in 2022, which is expected to reach US$353.9 billion annually by 2032, with a compound annual growth rate (CAGR) of 13.6%. In North America, consumer purchases from online pharmacies are projected to grow from US$39.4 in 2022 to US$138.9 in 2032, with a CAGR of 13.4% (Global Market Insights, 2023).

Online pharmacies include major corporations (e.g., Express Scripts Pharmacy, Walgreens, Amazon Pharmacy, and CVS), smaller independent pharmacies, and even illicit online pharmacies. Illicit online pharmacies violate regulations by selling counterfeit, adulterated, or unapproved drugs or dispensing prescription drugs without a valid prescription (Limbu and Huhmann, 2023a)

Research on online pharmacies has been growing. Reviews of online pharmacies show how the literature documents online pharmacy types and characteristics, the therapeutic classes and quality of drugs available, and risks to patients and public health (Orizio et al., 2011; Mackey and Nayyar, 2016; Long et al., 2022; Limbu and Huhmann, 2023a).

Despite a growing literature stream, a review has yet to be carried out to summarize and synthesize the studies that have examined factors predicting consumers’ online medicine purchase intentions and behavior. Only one review touches on consumer purchases (Almomani et al., 2023b). This narrative review of 17 studies identifies the prevalence of and reasons for prescription medicine purchases using any internet platform, including search engines, social media, and encrypted messaging applications; however, half the qualitative studies reviewed sampled pregnant women, which limits generalizability. Further, it solely focused on studies of prescription medication purchases published between 2012 and 2021. Although Almomani et al.’s (2023b) review included any internet purchases of prescription medications, its narrow search terms missed many studies of online pharmacy purchases. In addition, it included no studies published prior to 2012 or after 2021. Our study reviews almost three times as many studies on online pharmacy purchases alone.

Thus, to better understand why consumers increasingly purchase from online pharmacies, including illicit online pharmacies, the current scoping review rigorously examines all qualitative and quantitative studies of consumers’ online medication purchase intentions and behavior. Unlike earlier reviews, we included studies of consumer purchases of both prescription and over-the-counter (OTC) medications to determine if the need for a prescription alters why consumers want to acquire medication online. Also, we did not constrain our review to studies published within a limited date range. This allowed us to show how research into this topic has changed over time. Thus, this scoping review provides a comprehensive understanding of the nature of research into consumers’ online medication purchases.

Moreover, our review makes an important distinction between influences on consumer purchase intentions and influences on actual purchase behavior. Although some assume purchase intentions predict actual purchase behavior, prior research in many disciplines has found that they are not the same. In fact, meta-analyses have found that intentions account for only 28% of the variance in behavior and that a moderate-to-large change in intentions only results in a small-to-moderate change in behavior (Sheeran, 2002; Webb and Sheeran, 2006). Variables that increase or reduce the relationship between purchase intentions and behavior include unexpected situations, social influence, type of product, intervening time, environmental context, consumers’ self-efficacy, and the receipt of new information (Cote et al., 1985; Morwitz et al., 2007; Sheeran, 2002; Webb and Sheeran, 2006). For example, a consumer may intend to purchase from an online pharmacy, but time or social influence may shift that purchase to a brick-and-mortar pharmacy. Alternatively, a consumer may not intend to purchase from an online pharmacy, but an unexpected situation (e.g., a web search for information on a drug revealing a lower price at an online pharmacy) may encourage online medication purchase behavior.

The current review addresses three key research questions: (1) to what extent have consumer purchase intentions and behavior been investigated as they relate to online pharmacies? (2) how do the antecedents of consumers’ online purchases differ between OTC and prescription medications? and (3) what factors have been identified in the published literature that help explain consumers’ online pharmacy purchase intentions and behavior?

Hence, our investigation aims to fill a research gap by systematically scoping a body of literature on this topic of growing interest to researchers in healthcare and public policy. In addition, we provide guidance for the direction of future research and the areas that remain uninvestigated or unstudied, as well as those that represent emerging topics that warrant greater investigation.

## 2 Materials and methods

We carried out a scoping review, which is useful to map the literature on evolving or emerging fields or topics (Munn et al., 2018), such as the topic covered in the present study. This scoping review was coducted following the guidelines recommended by the Preferred Reporting Items for Systematic Reviews and Meta-Analyses (PRISMA), a commonly used reporting guidance for systematic reviews (Liberati et al., 2009). As such, this scoping review applies (Arksey and O’Malley, 2005) five-step framework: (1) research question identification, (2) relevant study identification, (3) study selection via inclusion and exclusion criteria, (4) data charting and investigation, and (5) result presentation.

### 2.1 Study identification

We searched PubMed, Web of Science, and Scopus to locate relevant articles. PubMed, one of the most commonly used search tools, is a leading database that covers biomedical and life sciences literature. Web of Science and Scopus are the two largest and most widely used bibliographic databases for searching multidisciplinary literature, as covered in our review.

To improve the retrieval of relevant articles, we used a wide range of search terms, such as online, internet, social media, pharmac*, buy or purchas*, and drug or medic* for either medication or medicine. To locate more records from this interdisciplinary literature, we also used relevant synonyms and variants of search terms. As presented in Table 1, we generated various search strings to execute exhaustive queries in each database by combining two or more search terms with the Boolean operators “AND” and “OR".

**TABLE 1:** Search stratetegy

Database	Search terms (Boolean operators)	#Records
PubMed	(((((online [Title) OR (internet [Title)) AND (pharmac*[Title)) AND (buy [Title)) OR (purchas*[Title)) AND (drug [Title))	426
	(((((online [Title) OR (internet [Title)) AND (pharmac*[Title)) AND (buy [Title)) OR (purchas*[Title)) AND (medic*[Title))	
Web of Science	(((((TI=(online)) OR TI=(internet)) AND TI=(pharmac*)) AND TI=(buy)) OR TI=(purchas*)) AND TI=(medic*) and Review Article (Exclude–Document Types) and Enriched Cited References and Article (Document Types) and Proceeding Paper (Exclude–Document Types) and English (Languages)	486
	(((((TI=(online)) OR TI=(internet)) AND TI=(pharmac*)) AND TI=(buy)) OR TI=(purchas*)) AND TI=(drug) and Article (Document Types) and English (Languages) and Article or Early Access (Document Types)	
	((((TI=(social media)) AND TI=(pharmac*)) AND TI=(buy)) OR TI=(purchas*)) AND TI=(drug)) and Review Article (Exclude–Document Types) and Enriched Cited References and Article (Document Types) and Proceeding Paper (Exclude–Document Types) and English (Languages)	
	((((TI=(social media)) AND TI=(pharmac*)) AND TI=(buy)) OR TI=(purchas*)) AND TI=(medic*)) and Review Article (Exclude–Document Types) and Enriched Cited References and Article (Document Types) and Proceeding Paper (Exclude–Document Types) and English (Languages)	
Scopus	(TITLE-ABS-KEY (online) OR TITLE-ABS-KEY (internet) OR TITLE-ABS-KEY (social AND media) AND TITLE-ABS-KEY (pharmac*) AND TITLE-ABS-KEY (buy) OR TITLE-ABS-KEY (purchas*) AND TITLE-ABS-KEY (drug) AND TITLE-ABS-KEY (medic*)) AND (LIMIT-TO DOCTYPE,“ar”)) AND (LIMIT-TO (LANGUAGE,“English”)) AND (LIMIT-TO (SRCTYPE,“j"))	258

### 2.2 Inclusion and exclusion criteria

**FIGURE 1:**
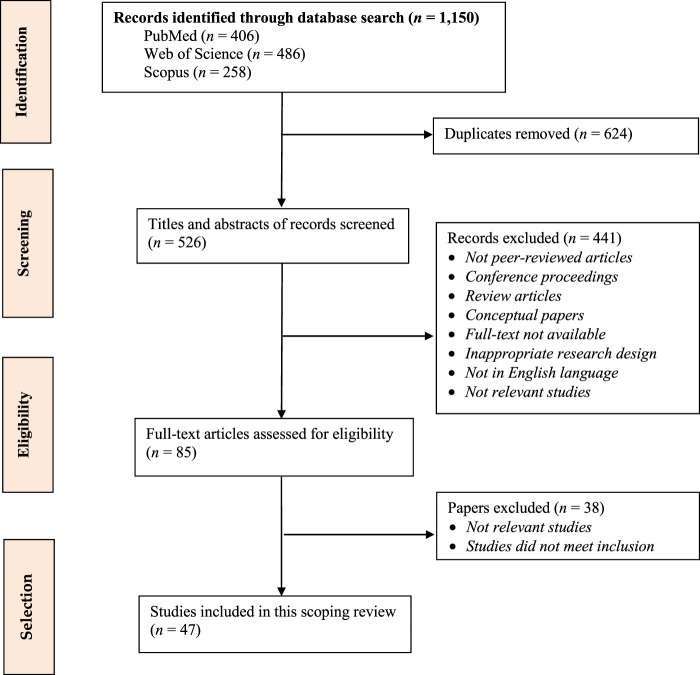
PRISMA flow diagram showing inclusion and exclusion of studies.

We limited the search criteria to exclude all literature irrelevant to our study, such as conference proceedings, grey literature, reviews, books, dissertations, and non-English articles. We included qualitative and quantitative studies of the determinants of medication purchase intentions and behavior over the Internet. Restrictions on the publication year and study population were not applied. The PRISMA flow diagram (see Figure 1) shows the article selection process, which consists of four steps (i.e., identification, screening, eligibility, and selection). It summarizes the number of records identified, screened, and excluded, the reasons for exclusion, and the number of studies included in this review (Liberati et al., 2009).

We retrieved 1,150 records from electronic databases, which consisted of 406 records from PubMed, 486 from Web of Science, and 258 from Scopus. After removing duplicates, 526 records were retained. Next, titles and abstracts were screened by two researchers independently. After screening titles and abstracts, 441 records that did not meet the inclusion criteria were eliminated. Finally, we assessed the remaining 85 full-text articles. Of these, 38 articles did not meet the eligibility criteria. Forty-seven studies were included in this scoping review.

### 2.3 Data extraction and analysis

All abstracts and full texts were screened and reviewed independently by two trained coders, who extracted data including authors, publication year, country, journal, population, sample size, research design, drug type, study objective, determinants of purchase intention and behavior, and key findings. Following Arksey and O’Malley (2005), the authors used an iterative method to synthesize the data by sorting prior research findings into key types of factors (i.e., data charting and investigation). IBM SPSS Statistics 27 was used to analyze the data.

## 3 Results

### 3.1 Description of included studies

This study included forty-seven studies from twenty-nine countries, including nine studies conducted in the United States, five studies in Saudi Arabia, and three studies each in Australia, China, India, and the United Kindom (see Table 2). Twenty-one studies were carried out in Asia, eighteen studies in Europe, eleven studies in North America, three studies in Australia, and two studies in Africa. Interestingly, no research was carried out in South America.

**TABLE 2:** Characteristics of studies included in this review.

Author(s)	Year	Country	Journal	Population	Sample size	Design	Drug
Abanmy	2017	Saudi Arabia	Saudi Pharmaceutical Journal	Adults	633	Survey	NS
Adjie et al	2023	Indonesia	Informatics in Medicine Unlocked	Adults	778	Survey	NS
Ahmed et al	2023	Bangladesh	International Journal of Healthcare Management	Adults	336	Survey	NS
Alfahad et al	2015	Saudi Arabia	Latin American Journal of Pharmacy	Adults	344	Survey	NS
Alhabash et al	2022	United States	International Journal of Environmental Research and Public Health	Adults	730	Experiment	Prescription
Almohammed et al	2023	Saudi Arabia	Saudi Pharmaceutical Journal	Adults	262	Survey	NS
Almomani et al	2023	UK	JMIR Formative Research	Adults	20	Interview	Prescription
Alsadoun et al	2023	Saudi Arabia	International Journal of Electronic Healthcare	Adults	NA	Survey	NS
Alwhaibi et al	2021	Saudi Arabia	Saudi Pharmaceutical Journal	Adults	643	Survey	Both
Anis and Tan	2023	Malaysia	International Journal of Healthcare Management	Adults	13	Interview	OTC
Ashames et al	2019	UAE	Journal of Pharmacy and Bioallied Sciences	Adults	528	Survey	Prescription
Bansal et al	2022	India	Indian Journal of Pharmacology	Adults	322	Survey	NS
Brijnath et al	2015	Australia	Patient	Adults	50	Interview	NS
Bowman et al	2020	Malta	Research in Social and Administrative Pharmacy	Adults	444	Survey	OTC
Brown and Li	2014	United States	Journal of the American Pharmacists Association	Adults	443	Survey	Prescription
Cicero and Ellis	2012	United States	Journal of Medical Internet Research	Patients	96	Survey	Prescription
Cokro and Arfenda	2023	Indonesia	Pharmacy Education	Students	95	Survey	OTC
Desai et al	2015	United States	Research in Social and Administrative Pharmacy	Adults	871	Survey	NS
Fittler et al	2018	Hungary	Journal of Medical Internet Research	Patients	1,055	Survey	NS
Fittler et al	2022	Czech Republic, Hungary, Poland, Slovakia	Frontiers in Pharmacology	Adults	2087	Survey	NS
Gharaibeh et al	2023	Jordan	BMJ Open	Adults	428	Survey	NS
Hamdan	2023	Jordan	Journal of Pharmaceutical Health Services Research	Adults	460	Survey	NS
Han and Han	2023	China	Frontiers in Public Health	Adults	414	Survey	NS
Hawdon et al	2022	United States	American Journal of Criminal Justice	Adults	1,101, 1,037, 1,250, 1,205	Longitudinal Survey	NS
Holiday-Goodman et al	2007	United States	Journal of Pharmacy Technology	Adults	91	Survey	Prescription
Holtgrafe and Zentes	2012	Germany	Health Informatics Journal	Adults	314	Survey	OTC
Hu et al	2021	China	Transformations in Business & Economics	Adults	202	Survey	OTC
Ivanitskaya et al	2010	United States	Journal of Medical Internet Research	Students	1914	Survey	Prescription
Jairoun et al	2021	UAE	Journal of Pharmaceutical Policy and Practice	Adults	131	Survey	Both
Koenraadt and van de Ven	2018	Netherlands	Drugs: Education, Prevention and Policy	Adults	158	Survey	Lifestyle drugs
Little et al	2020	Australia, Canada, Ireland, Portugal, Sweden, UK	Evidence Based Midwifery	Pregnant women	23	Focus group	NS
Ma	2021	China	Journal of Engineering and Technology Management	Adults	355	Survey	NS
Mekawie and Hany	2019	Egypt	Procedia Computer Science	Adults	210	Survey	OTC
Moureaud et al	2021	United States	Health Policy	MTurk workers	730	Survey	Prescription
Ndem et al	2019	Nigeria	Pharmacy Practice	Adults	500	Survey	NS
							
Rajamma and Pelton	2009	United States	Psychology & Marketing	Baby Boomers	350	Survey	Both
Roblek et al	2018	Slovenia	International Journal of Electronic Marketing and Retailing	Adults	378	Survey	OTC
Sabbir et al	2021	Bangladesh	Journal of Science and Technology Policy Management	Young people	285	Survey	NS
Sinclair et al	2018	UK	Journal of Advanced Nursing	Pregnant women	284	Survey	NS
Soboleva et al	2022	Russia	Journal of Advanced Pharmaceutical Technology & Research	Adults	3,789	Survey	NS
Srivastava and Raina	2020	India	International Journal of Pharmaceutical and Healthcare Marketing	Adults	184	Survey	NS
Szekely et al	2015	Romania	Farmacia	Patients	253	Survey	NS
Tang et al	2023	Japan	Frontiers in Digital Health	Adults	288	Survey	OTC
Van Buskirk et al	2016	Australia	International Journal of Drug Policy	Adults	66	Survey	NS
Varghese Assin et al	2022	India	Special Education	Adults	750	Survey	NS
Vera-Martínez	2023	Mexico	International Journal of Pharmaceutical and Healthcare Marketing	Adults	271	Survey	NS
Wiedmann et al	2010	Germany	Journal of Customer Behaviour	Adults	152	Interview	NS

Note: OTC, over the counter; Both = prescription and OTC; NS, not specified.

All studies, except a longitudinal study by Hawdon et al. (2022), were cross-sectional. Forty-two studies employed survey methodology, four interviewed adult individuals, one used a focus group, and another conducted an experiment Three studies were published in the *Journal of Medical Internet Research*, and three appeared in the *Saudi Pharmaceutical Journal*. The included studies recruited 27,323 respondents, with an average sample size of 581.34 (standard deviation = 651.23), ranging from 13 to 3,789. Thirty-seven studies focused on the general adult population, three on patients, two on women, two on students, and three on other populations. Eight studies focused on prescription drugs, eight studies on OTC medication, and the remainder either included both or did not specify.

**FIGURE 2:**
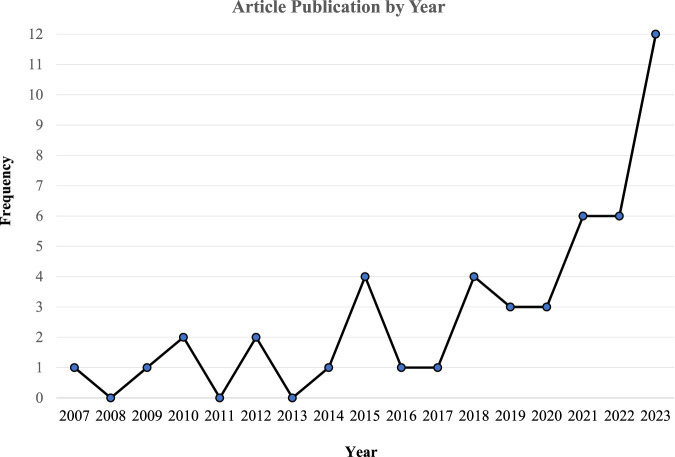
Number of studies by year.

Figure 2 shows consistently few studies conducted on the topic until 2014. Four studies were published in 2015, then the publications sharply fell in 2016 and 2017. Beginning in 2018, we see a growing wave of publications peaking in 2023, with twelve studies.

### 3.2 Differences in influences between OTC and prescription drugs

Our analysis of prior studies revealed some differences between OTC and prescription medication in factors influencing online purchase intentions and behaviors. Intentions toward online purchases of prescription medications were primarily motivated by price, availability, convenience, and education. Prescription drug online purchase behavior was adversely affected by perceived risk. Purchase behavior was positively related to health insurance coverage, information sources, and convenience.

Most studies involving OTC medication studied purchase behavior. The major driving forces of consumer purchases of OTC medication online were information sources, availability, internet usage, and convenience. Evaluation of the purchase environment, information sources, and prior experience primarily influenced purchase intentions toward OTC medication online.

### 3.3 Factors influencing purchase intention and behavior from online pharmacies

#### 3.3.1 Demographics

As shown in Table 3, education, gender, and income were the most frequent demographic predictors of purchase intentions toward online pharmacies. However, the scoping review reveals some contradictory findings. Higher education was at times associated with increased purchase intentions (Holiday-Goodman et al., 2007; Sinclair et al., 2018; Hu et al., 2021). In contrast, another study indicated that individuals with lower education levels had greater intentions to purchase from an online pharmacy (Ivanitskaya et al., 2010). Holiday-Goodman et al. (2007) found that American males had greater intentions toward online pharmacy purchases than females. In contrast, Hu et al. (2021) showed that Chinese females had greater online purchase intentions for OTC medications online than did males. Higher incomes increased consumers’ online medicine purchase intentions (Holiday-Goodman et al., 2007; Hu et al., 2021). Online pharmacy purchase intentions also were greater among young, White, and married consumers (Holiday-Goodman et al., 2007).

**TABLE 3:** Factors influencing purchase intention and behavior from online pharmacies.

Factors	Intention	Behavior
Demographics	Education (higher) Holiday-Goodman et al. (2007); Sinclair et al. (2018); Hu et al. (2021)	Age (older) Brown and Li, (2014); Desai et al. (2015); Jairoun et al. (2021); Soboleva et al. (2022); Cokro and Arfenda, (2023)
	Education (lower) Ivanitskaya et al. (2010)	Age (younger) Szekely et al. (2015); Van Buskirk et al. 2(016); Vera-Martínez, (2023)
	Gender (male) Holiday-Goodman et al. (2007)	Education (higher) Rajamma and Pelton, (2009); Brown and Li, (2014); Roblek et al. (2018)
	Gender (female) Hu et al. (2021)	Education (high school) Jairoun et al. (2021)
	Income (higher) Holiday-Goodman et al. (2007); Hu et al. (2021)	Education (high school or university) Szekely et al. (2015)
	Age (younger) Holiday-Goodman et al. (2007)	Gender (male) Rajamma and Pelton, (2009); Szekely et al. (2015); Jairoun et al. (2021)
	Race (White) Holiday-Goodman et al. (2007)	Gender (female) Soboleva et al. (2022)
	Marital status (married) Holiday-Goodman et al. (2007)	Income (higher) Rajamma and Pelton, (2009); Brown and Li, (2014); Desai et al. (2015); Roblek et al. (2018)
		Race (White) Brown and Li, (2014)
		*Race (Black) Brown and Li, (2014)*
		Marital status (married) Brown and Li, (2014)
		Marital status (single) Jairoun et al. (2021)
		Location (urban area) Roblek et al. (2018)
Convenience	Fast delivery, choices of delivery times and address, reduced visit to the pharmacy, easy telephone access to the pharmacist for drug information Alfahad et al. (2015)	Convenience Brijnath et al. (2015); Abanmy, (2017); Koenraadt and van de Ven, (2018); Little et al. (2020); Almomani et al. (2023); Anis and Tan, (2023); Tang et al. (2023)
	Perceived ease of use Ahmed et al. (2023)	Time saving Almohammed et al. (2023); Almomani et al. (2023)
	Home delivery Ahmed et al. (2023)	Easy access Alwhaibi et al. (2021); Almomani et al. (2023)
	Accessibility (cannot find a doctor who will prescribe, doctor will not prescribe enough, no other way to get it) Cicero and Ellis, (2012)	Bypassing gatekeepers Almomani et al. (2023)
	Convenience Holiday-Goodman et al. (2007)	Home delivery Koenraadt and van de Ven, (2018)
	Consumer is a convenience-oriented rationalist toward online pharmacies Wiedmann et al. (2010)	Importance of rapid retrieval of information Little et al. (2020)
		Price comparison Little et al. (2020)
		Time-efficiency Little et al. (2020)
Availability	Unavailable in local market Sinclair et al. (2018); Ashames et al. (2019); Bansal et al. (2022)	Availability of various drug products and more options Little et al. (2020); Cokro and Arfenda, (2023)
	Ability to get legitimate medicine from online pharmacies Moureaud et al. (2021)	Unavailable in local market Abanmy, (2017); Bowman et al. (2020)
		Wide-spread availability Tang et al. (2023)
		Not for sale in the country Koenraadt and van de Ven, (2018))
		24-h availability Tang et al. (2023)
Price	Differences in the prices Bansal et al. (2022)	Lower price Brijnath et al. (2015); Van Buskirk et al. (2016); Abanmy, (2017); Koenraadt and van de Ven, (2018); Bowman et al. (2020); Alwhaibi et al. (2021); Almohammed et al. (2023); Almomani et al. (2023)
	Lower price Cicero and Ellis, (2012); Sinclair et al. (2018); Ashames et al. (2019); Adjie et al. (2023)	Cost-effectiveness Little et al. (2020)
	Price value Han and Han, (2023)	*Higher price* Almomani et al. (2023)
	Deals Moureaud et al. (2021)	Offers and discounts Almohammed et al. (2023)
		Special offers Little et al. (2020)
Evaluations of purchase environment	Attitude toward online pharmacies Hu et al. (2021); Han and Han, (2023)	Trust in buying OTC drugs online Roblek et al. (2018)
	View pharmacy website design as attractive and useful Hu et al. (2021)	Trust in purchasing drugs from online resources Gharaibeh et al. (2023)
	Emotional response to online pharmacies Hu et al. (2021)	Trust in online pharmacy websites Almomani et al. (2023)
	Attitude toward online pharmacy logistics and distribution Hu et al. (2021)	Perceived benefits of online drug purchasing Gharaibeh et al. (2023)
	Perceived usefulness of online pharmacies Ma, (2021)	
	Perceived behavioral control Han and Han, (2023)	
	Trustworthiness of online pharmacies Ma, (2021); Sabbir et al. (2021)	
	Trust in e-marketplace Adjie et al. (2023)	
	Trust in website technology Alsadoun et al. (2023)	
	Perceived trust in app-based medicine service Ahmed et al. (2023)	
	Perceived website system and service quality Adjie et al. (2023)	
	Online pharmacies provide a reliable source for purchases Moureaud et al. (2021)	
	Effort expectancy (usage of online pharmacy is simple, clear and justifiable) Srivastava and Raina, (2020); Sabbir et al. (2021)	
	Facilitating conditions (organizational and technical infrastructures assist consumers to use online pharmacy) Sabbir et al. (2021)	
	Performance expectancy (i.e., usefulness of online pharmacies) Srivastava and Raina, (2020); Sabbir et al. (2021)	
	Hedonic motivation (i.e., enjoy using online pharmacies) Srivastava and Raina, (2020)	
	Satisfaction with the e-marketplace Adjie et al. (2023)	
Internet usage	Time spent on internet Fittler et al. (2018)	Frequency of internet usage Roblek et al. (2018)
		Frequency of usage of social media Roblek et al. (2018)
		Preferred use of the internet as a source of OTC drug information Holtgrafe and Zentes, (2012)
		Information surfers (people who gather information on the internet) Vera-Martínez, (2023)
Information sources	Word-of-mouth (WOM) online Lee et al. (2017) e-WOM (sharing on Facebook, comment posting on Facebook, Facebook friends’ likes) Mekawie and Hany, (2019)	Word-of-mouth from friends Almomani et al. (2023)
	Drug information provided on website Hu et al. (2021)	Social media marketing Anis and Tan, (2023)
	Information quality on e-pharmacy websites Adjie et al. (2023)	Influencers’ endorsement Almomani et al. (2023)
	Recommended by family, friend, advertisers, or cultural sources Hu et al. (2021)	Information provided by suppliers Cokro and Arfenda, (2023)
	Recommendations from others Moureaud et al. (2021)	Consumers’ reviews Almomani et al. (2023); Anis and Tan, (2023)
	Social influence (influence from friends and families) Srivastava and Raina, (2020); Sabbir et al. (2021)	Interactions with healthcare providers Almomani et al. (2023); Desai et al. (2015)
	Subjective norms Adjie et al. (2023); Han and Han, (2023)	
		Recommendations from experts or non-experts Anis and Tan, (2023)
		*Powerful others health locus of control* (influence of healthcare provider) Rajamma and Pelton, (2009)
Perceived risk	*Perceived risk* Hu et al. (2021)	*Medicine safety concerns* Almomani et al. (2023)
	*Perceived risk (financial risk, physical risk, source risk)* Varghese Assin et al. (2022)	*Medicine quality concerns* Almomani et al. (2023)
		*Web-based payment* Almomani et al. (2023)
		*Lack of accountability* Almomani et al. (2023)
		*Engaging in an illegal behavior* Almomani et al. (2023)
Prior experience	Prior experience of purchase good online Fittler et al. (2018); Ndem et al. (2019)	Personal experience Anis and Tan, (2023)
	Comfort with the network transaction environment Hu et al. (2021)	
	Years of experience with online shopping Hu et al. (2021)	
	Greater health literacy (ability to find, understand, and use information needed to purchase from online pharmacies) Sabbir et al. (2021)	
	Lower electronic health literacy Ivanitskaya et al. (2010)	
	Awareness of online pharmacy website technology Alsadoun et al. (2023)	
	Consumer is an enthusiastic expert about online pharmacies Wiedmann et al. (2010)	
Health insurance	No healthcare coverage Cicero and Ellis, (2012)	Have higher health care expenditures Brown and Li, (2014)
		More likely to be privately insured Brown and Li, (2014)
		*Medicaid insurance coverage* Brown and Li, (2014)
		Health insurance coverage Desai et al. (2015)
Privacy	Anonymity Cicero and Ellis, (2012)	More privacy Alwhaibi et al. (2021); Almomani et al. (2023)
		Do not want to discuss with doctor Koenraadt and van de Ven, (2018)
Product	Product quality Holiday-Goodman et al. (2007); Van Buskirk et al. (2016)	Product varieties Alwhaibi et al. (2021); Almohammed et al. (2023)

Note: Italicized text indicates reported findings that are negatively related to purchase intention and behavior.

The most frequent demographic predictors of purchase behavior included age, education, gender, and income. Again, the evidence is contradictory. Five studies reported that more older consumers purchased from online pharmacies (Brown and Li, 2014; Cokro and Arfenda, 2023; Desai et al., 2015; Jairoun et al., 2021; Soboleva et al., 2022), whereas three studies found the opposite (Szekely et al., 2015; Van Buskirk et al., 2016; Vera-Martínez, 2023). In many studies, higher education was associated with increased purchase (e.g., Rajamma and Pelton, 2009; Brown and Li, 2014; Szekely et al., 2015; Roblek et al., 2018); however, Jairoun et al. (2021) found that consumers with less education were more likely to purchase medicine online. Three studies showed that males purchased medications online more frequently than females (Rajamma and Pelton, 2009; Szekely et al., 2015; Jairoun et al., 2021), whereas another study reported females as more likely to purchase online than males (Soboleva et al., 2022). Higher income was positively associated with increased medication purchases online (Rajamma and Pelton, 2009; Brown and Li, 2014; Desai et al., 2015; Roblek et al., 2018). Brown and Li (2014) found that black patients were associated with 53% lower odds of online pharmacy purchases than white patients. Brown and Li (2014) also reported that online pharmacy users were more likely to be married; however, Jairoun et al. (2021) found that single consumers purchased medication online more often than married consumers during the COVID-19 pandemic. OTC medicine was most often purchased online by young consumers living in urban areas than smaller settlements (Roblek et al., 2018).

#### 3.3.2 Convenience

Convenience was a frequently demonstrated predictor of purchase intentions (Holiday-Goodman et al., 2007). Convenience-related factors, such as fast delivery, choices of delivery times and addresses, reduced visits to the pharmacy, and easy telephone access to the pharmacist for drug information were common reasons supporting intentions to purchase medications from online pharmacies (Alfahad et al., 2015). Other convenience-related factors that enhanced consumers’ willingness to buy medicine online were perceived ease of use of app-based medical services (Ahmed et al., 2023), home delivery (Ahmed et al., 2023), and accessibility of prescription medications (e.g., cannot find a physician who will prescribe, the physician will not prescribe enough, or no other way to get it) (Cicero and Ellis, 2012).

Convenience was also a dominant motivation for purchasing behavior. Consumers who bought medications online indicated that buying drugs online was more convenient (Brijnath et al., 2015; Abanmy, 2017; Koenraadt and van de Ven, 2018; Little et al., 2020; Almomani et al., 2023; Anis and Tan, 2023; Tang et al., 2023) and saved time (Almohammed et al., 2023; Almomani et al., 2023). Other convenience-related motivations included easy access (Alwhaibi et al., 2021; Almomani et al., 2023), bypassing gatekeepers (Almomani et al., 2023), home delivery (Koenraadt and van de Ven, 2018), the importance of rapid retrieval of information (Little et al., 2020), price comparison (Little et al., 2020), and time efficiency (Little et al., 2020).

#### 3.3.3 Availability

Three studies reported that medication unavailability in the local market increased consumers’ intention to purchase over the Internet (Sinclair et al., 2018; Ashames et al., 2019; Bansal et al., 2022). Moureaud et al. (2021) found that getting legitimate medication was the most common reason that consumers intended to purchase medicine online.

The studies included in this review reveal that the availability of various drug products and more options (Little et al., 2020; Cokro and Arfenda, 2023), wide-spread availability (Tang et al., 2023), and 24-h availability (Tang et al., 2023) were primary motivators for purchasing medication online. Unavailability in the local market (Abanmy, 2017; Bowman et al., 2020) and drugs not for sale in a country (Koenraadt and van de Ven, 2018) were also positively related to increased purchase behavior.

#### 3.3.4 Price

Pricing factors enhancing consumers’ purchase intentions toward drugs online included lower prices (Cicero and Ellis, 2012; Sinclair et al., 2018; Ashames et al., 2019; Adjie et al., 2023), differences in the prices (Bansal et al., 2022), and price value, which is the perceived benefits given the cost (Han and Han, 2023).

Several studies indicated that price significantly promoted medication purchases online. Nine studies found that lower prices were positively associated with increased purchase behavior (Brijnath et al., 2015; Van Buskirk et al., 2016; Abanmy, 2017; Koenraadt and van de Ven, 2018; Bowman et al., 2020; Little et al., 2020; Alwhaibi et al., 2021; Almohammed et al., 2023; Almomani et al., 2023). Similarly, another study found that higher prices were associated with decreased purchase behavior (Almomani et al., 2023).

In addition, some studies explored the effects of sales promotions, which reduced prices. Sales promotions such as deals, discounts, and special offers were common facilitators of intentions to purchase medicine online (Little et al., 2020; Moureaud et al., 2021; Almohammed et al., 2023).

#### 3.3.5 Evaluations of purchase environment

Several studies examined the effects of consumers’ evaluations of the online medication purchase environment. Some purchase environment evaluations that enhanced consumers’ intentions to buy online included attitudes toward online pharmacies (Hu et al., 2021; Han and Han, 2023), emotional response to online pharmacies (Hu et al., 2021), attitude toward online pharmacy logistics and distribution (Hu et al., 2021), perceived usefulness of online pharmacies (Ma, 2021), perceived behavioral control (Han and Han, 2023), trustworthiness of online pharmacies (Ma, 2021; Sabbir et al., 2021), trust in the e-marketplace (Adjie et al., 2023), trust in website technology (Alsadoun et al., 2023), perceived trust in app-based medicine services (Ahmed et al., 2023), perception of pharmacy website design (Hu et al., 2021), perception of website system and service quality (Adjie et al., 2023), perception of online pharmacies as a reliable source for purchases (Moureaud et al., 2021), effort expectancy (i.e., online pharmacy is simple, clear and justifiable to use) (Srivastava and Raina, 2020; Sabbir et al., 2021), facilitating conditions (i.e., organizational and technical infrastructures assist consumers to use online pharmacy) (Sabbir et al., 2021), performance expectancy (i.e., usefulness of online pharmacies in achieving desired outcome) (Srivastava and Raina, 2020; Sabbir et al., 2021), hedonic motivation (i.e., enjoy using online pharmacies) (Srivastava and Raina, 2020), and satisfaction with the e-marketplace (Adjie et al., 2023).

Few studies explored purchase environment evaluations as determinants of purchase behavior. Consumers’ trust in buying OTC drugs online (Roblek et al., 2018), trust in purchasing drugs from online resources (Gharaibeh et al., 2023), trust in online pharmacy websites (Almomani et al., 2023), and perceived benefits of online drug purchasing (Gharaibeh et al., 2023) were positively associated with increased medication purchases online.

#### 3.3.6 Internet usage

One study showed that people who spent more time online were more willing to purchase medication online (Fittler et al., 2018). Purchase behavior was influenced by frequency of internet usage (Roblek et al., 2018) and information-seeking behavior on the internet (Vera-Martínez, 2023) as well as frequency of social media usage (Roblek et al., 2018) and use of the internet as a source of OTC drug information (Holtgrafe and Zentes, 2012).

#### 3.3.7 Information sources

Consumers’ intention to purchase medication online was enhanced by several information sources, including online word-of-mouth (Lee et al., 2017); social media word-of-mouth, such as sharing on Facebook and comments posted on Facebook (Mekawie and Hany, 2019); drug information provided on the website (Hu et al., 2021); information quality on e-pharmacy websites (Adjie et al., 2023); recommendations from family, friends, advertisers, or cultural sources (Hu et al., 2021); recommendations from others (Moureaud et al., 2021); social influence, such as interactions with or persuasion by friends and families (Srivastava and Raina, 2020; Sabbir et al., 2021); and subjective norms, which is the perceived social pressure or expectations regarding a behavior (Adjie et al., 2023; Han and Han, 2023).

Some information source-related factors enhancing purchase behavior included word-of-mouth from friends (Almomani et al., 2023), social media marketing (Anis and Tan, 2023), influencers’ endorsements (Almomani et al., 2023), information provided by suppliers (Cokro and Arfenda, 2023), reviews from other consumers (Almomani et al., 2023; Anis and Tan, 2023), and interactions with healthcare providers (Desai et al., 2015; Almomani et al., 2023).

#### 3.3.8 Perceived risk

Perceived risk associated with the online purchase of drugs, such as privacy concern and uncertainty of drug quality, negatively impacts consumers’ purchase intentions (Hu et al., 2021). Three dimensions of perceived risk -- that is financial, physical, and source risks (i.e., concern about the credibility and reliability of online pharmacies) -- adversely affected consumers’ purchase intentions towards online pharmacies (Varghese Assin et al., 2022).

Consumers’ purchase behavior was negatively influenced by medicine safety concerns (Almomani et al., 2023), medicine quality concerns (Almomani et al., 2023), web-based payment (Almomani et al., 2023), lack of accountability (Almomani et al., 2023), and concerns that online purchases constitute engagement in illegal behavior (Almomani et al., 2023).

#### 3.3.9 Prior experience

Research shows that prior experience of purchasing goods online (Fittler et al., 2018; Ndem et al., 2019); online shopping experience (Hu et al., 2021); comfort with the network transaction environment (Hu et al., 2021); greater health literacy, that is ability to find, understand, and use information needed to purchase from online pharmacies (Sabbir et al., 2021); lower electronic health literacy (Ivanitskaya et al., 2010); awareness of online pharmacy website technology (Alsadoun et al., 2023); and consumers’ expertise regarding online pharmacies (Wiedmann et al., 2010) were positively associated with purchase intentions. Personal experience also influenced OTC medication purchase behavior (Anis and Tan, 2023).

#### 3.3.10 Health insurance

Research showed that people without health insurance coverage were more likely to demonstrate purchase intentions toward online pharmacies (Cicero and Ellis, 2012). Similarly, another study reported an inverse association between Medicaid insurance coverage and purchase behavior (Brown and Li, 2014). However, two other studies found that health insurance coverage motivated consumers to engage in online medication purchase behavior (Brown and Li, 2014; Desai et al., 2015).

#### 3.3.11 Privacy

Cicero and Ellis (2012) showed that perceived anonymity could be a primary reason that consumers intended to purchase from online pharmacies that dispensed Tramadol, a prescription medication, without a prescription. In terms of behavior, Alwhaibi et al. (2021) indicated that consumers are motivated to purchase online as it provides greater assurance of confidentiality and access to medicine. Similarly, consumers’ perceptions of privacy can influence their decision to buy some prescription medicines online (Almomani et al., 2023) as they may avoid embarrassment by obtaining their medicines without the need for face-to-face interactions or to state their illness when the shame of admitting a condition acts as a barrier to seeking care (e.g., disclosing sexual dysfunction or seeking slimming pills for weight loss). For example, many consumers bought sexual enhancers online because they did not want to discuss their condition with their physician (Koenraadt and van de Ven, 2018).

#### 3.3.12 Product

As shown in Table 3, two studies reported a positive association between product quality and behavioral intentions (Holiday-Goodman et al., 2007; Van Buskirk et al., 2016). In two other studies, product variety motivated purchase behavior from online pharmacies (Alwhaibi et al., 2021; Almohammed et al., 2023).

## 4 Discussion and implications

This scoping review sought to understand the current landscape of empirical research on the drivers of consumer purchase intentions and usage behavior of online pharmacies. Scholarly interest in this topic has been growing exponentially over the last few years. Using rigorous scoping review methods, forty-eight articles published to date met the inclusion criteria, including 12 published in 2023.

Although we sought qualitative and quantitative studies that applied various methodological approaches, the existing literature was mostly limited to surveys. In terms of sample populations, however, the existing literature exhibits greater diversity in both population types (e.g., adults, patients, pregnant women) and geographic areas, such as Europe (e.g., Germany, Hungary, Ireland, Malta, Netherlands, Portugal, Romania, Russia, Slovenia, Sweden, and the UK), Asia (e.g., Bangladesh, India, China, and Taiwan), North America (Canada and the United States), the Arabian Peninsula (e.g., Saudi Arabia and the United Arab Emirates), Africa (e.g., Egypt and Nigeria), and Australia. However, Latin America has been ignored in the existing literature, including the major markets of Mexico, Brazil, Chile, Colombia, and Argentina.

This scoping review identified twelve general factors, which include many individual facilitators and inhibitors of consumer purchase intentions and behavior that have been studied so far (Table 3). Of these, the greatest amount of research has investigated the demographics of consumers who purchase or intend to purchase from online pharmacies. Due to the conflicting results, few implications can be gleaned from the prior research related to demographics or the role of health insurance coverage. However, higher-income consumers, White American consumers, and more educated consumers (in nine of ten studies) had higher online medication purchase intentions and behavior. The implication for researchers and practitioners from the income and education findings seems to be that greater financial and cognitive resources help consumers navigate the online environment to find medications for purchase. Policymakers seeking to dissuade purchases from rogue or illegitimate online pharmacies should target messages and campaigns to these demographic groups.

Convenience and availability factors more consistently influenced online medication purchase intentions and behavior. Consumers intended or chose to purchase from online pharmacies due to faster and more convenient delivery, ease of use and gathering information, accessibility, the ability to bypass healthcare providers or regulatory gatekeepers, availability outside of store hours, and access to medications unavailable locally. A practical implication for brick-and-mortar pharmacies is that enhancing their convenience (e.g., same day delivery, faster service at the pharmacy counter, or longer hours of operation) should help them better compete with online pharmacies.

Price was also a consistent influence on online medication purchase intentions and behavior. Factors that lowered prices or increased price savings and value received from online medicine purchases tended to attract consumers. Thus, a practical implication is that online pharmacies should emphasize price savings to continue to grow their share of consumers’ drug purchases. To better challenge online pharmacies, brick-and-mortar pharmacies should become more price competitive. A policy implication is that public policymakers and regulators attempting to control the illicit dispensing of medicine online should consider regulation reductions, subsidies, or other ways to lower prices through legitimate pharmacies.

Favorable evaluations of the purchase environment also consistently benefitted purchase intentions and behavior across many types of evaluations, such as attitudes, trust, emotional response, aesthetically pleasing and functional design evaluations, enjoyment, and satisfaction. Thus, it appears that many consumers who purchase from or intend to purchase from online pharmacies do so because they hold positive evaluations of those pharmacies. An important implication of this finding is that policymakers, regulators, and brick-and-mortar pharmacies interested in discouraging online medication purchases must engage in attitude change campaigns before expecting consumers who purchase online to alter their intentions or behavior. Attitude change can take time as it requires learning new information and responses through repeated exposure to counterinformation or social influence from family, friends, or trusted others, such as experts or celebrities.

This scoping review summarized prior research findings in which social influence and other information sources motivate online medication purchase intentions and behavior. Intentions and behavior were influenced by information and recommendations from expert sources, such as healthcare providers and websites with drug information; personal sources, such as friends and family; independent sources, such as social media influencers and reviews by other consumers; and non-personal sources, such as social media marketing. One implication is that pharmacists, healthcare providers, policymakers, and regulators should be able to indirectly influence consumer behavior toward online medicine purchases if they tap into the social and normative influence of one or more of these information sources.

Perceived financial, physical, or source risks inhibit online medicine purchase intentions and behavior, whereas greater health literacy and experience with online pharmacies, online shopping in general, and the internet or social media encourage online medicine purchasing. The benefits of online pharmacies (e.g., greater perceptions of anonymity or privacy, better perceived product quality, and more product variety) also encourage online medicine purchase intentions and behavior. An implication is that brick-and-mortar competitors or public policymakers should heighten consumer awareness of these risks if they wish to discourage consumer online medicine purchases. Further, legitimate online sellers can highlight risks to differentiate their verified online pharmacies from illicit or rogue online pharmacies.

Finally, this scoping review documented other factors that had been investigated sparingly (see Appendix A). For example, some studies have begun to investigate how consumer personality predisposes some consumers toward (e.g., greater purchase involvement, variety seeking, and openness) or against (e.g., objectivism) online medicine purchasing. Other studies have begun to investigate situational influences on consumer online medication purchase intentions and behavior (e.g., the COVID-19 pandemic). Individually, these other factors have been studied so rarely that it is difficult to confidently predict the general effect on purchase intentions and behavior without future research. These other influences are listed in the Appendix A.

## 5 Directions for future research

The analyzed literature indicates several gaps in the literature and future research directions that could be addressed. First, although this scoping review covers the vast majority of the literature published in English to date on consumers’ online medication purchase intentions and behavior, research interest in this topic is growing exponentially, with several new studies published each year. Thus, a systematic review or meta-analysis that incorporates the post-2023 studies will likely be able to be conducted within the next decade once sufficient research has been published. This should further our understanding of the factors that facilitate and inhibit online medication purchasing as well as help determine the relative strength of different influences.

Second, this scoping review found that the current state of research into the drivers of consumers’ online medication purchase intentions and behavior is methodologically limited. Most studies (87.5%) use a survey design. The remainder includes a handful of qualitative studies and a single experiment. Future research employing methods other than surveys (e.g., experiments, observational methods, and data analytics) can investigate research topics that are difficult to study via self-report methods. Similarly, future research should advance the understanding of online purchase drivers by using more generalizable and diverse samples, patient rather than general adult samples, and samples that permit cross-cultural comparisons. Future research could also investigate more complex models of potential drivers’ relationships with purchase intentions and/or behavior, such as mediation or moderation models that could resolve the conflicting evidence in prior research that this scoping review has uncovered and identify important boundary conditions on these effects.

Third, this scoping review finds that a vast majority of the prior research did not specify whether online purchases of OTC or prescription medication or both were being studied. Future research should specify this because the current review shows differences in the drivers of online OTC and prescription drug purchase intentions and behavior. In fact, due to the paucity of literature on this issue, future research is needed to more confidently delineate the drivers of consumers’ online purchase intentions and behavior for OTC *versus* prescription drugs.

Fourth, demographics (e.g., consumers’ age, gender, or educational attainment) are easy to measure but appear to be poor predictors of purchase intentions or behavior, given the large number of conflicting results related to demographic variables in the studies reviewed. As noted in previous reviews (Tugwell et al., 2008; Schmid et al., 2017; Limbu and Huhmann, 2023c), demographic variables are carrier variables that are not mechanisms causing changes in intentions or behavior but are instead associated to some degree with the prior experiences, predispositions, beliefs, attitudes, or characteristics that affect consumer response. Thus, rather than relying on demographics, future research should attempt to identify the underlying explanatory determinants to better predict purchase intentions and behavior from online pharmacies. Studies, including moderation or mediation tests, could also help better understand the mechanisms at work. Once the underlying explanatory determinants associated with a demographic variable such as age or gender have been identified, the indirectly related demographic variables can serve to identify likely target audiences for public health campaigns and patient education materials regarding online pharmacies.

Fifth, although this scoping review documents many influences that increase or decrease consumers’ purchase intentions and/or behavior, it also underscores the need for future research into uninvestigated and understudied influences. For example, psychographic variables, such as personality, perceptions, attitudes, beliefs, lifestyle, interests, and values, can offer the potential to identify deeper motivations and could better identify why some consumers buy medications online whereas others do not. To direct future research into other uninvestigated or underinvestigated areas, Table 4 lists some research questions that warrant exploration to better understand the driving influences on consumer purchase intentions and behavior.

**TABLE 4:** Future research agenda and research questions.

Drivers	Concepts/theories	Potential research questions
Risk	Perceived risk	How do differences in the degree of risk across specific medications, dosages, and efficacies impact consumers’ intentions to purchase online or their actual purchase behavior?
		To what degree do consumers perceive online purchases of medicine to involve different types of risk (e.g., financial or physical)? How much financial risk do they perceive and why (e.g., fraudulent charges after online medicine purchases or waste of money on ineffectual/fake drugs)? How much physical risk do they perceive and why (e.g., fake medicine purchased online harms health or is adulterated with opioids leading to addiction)? How much source risk do consumers perceive and why (e.g., personal information for online medication purchase used in spam emails/calls, invasion of privacy, or identity theft)?
		What is the nature and prevalence of adverse effects suffered by consumers who purchase medicine online? Does it differ across types of online pharmacies (e.g., chain, independent, and cost-plus legitimate pharmacies *versus* rogue/illicit pharmacies)?
	Uncertainty avoidance	How do consumers attempt to manage the risk of online medical purchases?
		How can public policymakers highlight the risks of illicit or rogue pharmacies in educational campaigns to decrease consumer purchases?
	Decision-making heuristics	What heuristics and cues do consumers use to identify “safe” online medicine sources for purchase? Are these heuristics and cues accurate?
	Regulatory focus theory	What risks do medicine purchases online pose to healthcare providers? How prepared are they to address these risks, and how can educators better prepare medical and pharmaceutical students to better address the risks as future healthcare practitioners who are involved in monitoring and discussing online sources of medicine with patients?
		How do consumers’ online medication purchase intentions and behavior differ if they are primarily prevention-focused (focused on avoiding losses) or promotion-focused (focused on seeking gains)?
Benefits	Manifest and latent motives	How do the manifest motives (benefits that consumers freely admit seeking) differ from the latent motives (those that consumers do not want to admit or reveal to others) for online medication purchases?
	Benefits segmentation	How are consumers’ online medicine purchase intentions and behavior impacted by the specific benefit sought (e.g., perceived anonymity, avoidance of embarrassment, cost savings, improved availability, greater autonomy, ability to self-diagnose/self-medicate, greater access, delivery to remote areas, or off-label usage)? How can traditional brick-and-mortar or legitimate online pharmacies better incorporate these benefits, and would doing so reduce consumer purchases from illicit online pharmacies? To what extent do consumers intend to purchase or purchase medicine specifically from illicit online pharmacies to seek these perceived benefits?
	Product features, attributes, and benefits	What benefits do consumers believe online medical purchases offer based on online pharmacies’ features and tangible attributes?
		How have closures of locations of large chain pharmacies (e.g., Rite-Aid and Walgreens) impacted consumers’ perceptions of online pharmacies offering benefits related to availability and convenience? Have such closures increased purchases from legitimate and illicit online pharmacies?
Learning	Information processing	How is the proportion of consumers who intend to or have purchased medicine online affected by the degree of awareness of online sources of medicine or by information about them stored in memory?
		What types of information increase *versus* overcome consumer resistance to purchasing medications online?
		How can education campaigns best model the dangers of illicit online pharmacies to discourage consumer purchases from them?
		How does providing information about online pharmacies, their risks, or benefits by public health officials in public service advertising campaigns impact consumer purchase intentions and behavior?
	Vicarious learning/modelling	How and to what extent does exposure to other consumers’ ratings and reviews available on online pharmacies impact consumer purchase intentions and behavior? How do these consumer ratings and reviews compare to healthcare providers’ professional opinions regarding outcomes, such as efficacy or side effects?
	Cognitive learning	What beliefs do consumers form based on exposure to other consumers’ ratings and reviews *versus* FDA-approved patient labeling?
		What types of information do healthcare providers communicate about purchasing medications online? How does this impact consumer beliefs, attitudes, and purchase intentions? What information would consumers find most useful to optimize purchase decisions?
	Cognitive capacity	Consumers’ cognitive resources are limited; how does this impact their acquisition and retention of information from various sources (e.g., healthcare providers, FDA-approved patient labeling, or online ratings and reviews) and subsequent purchase intentions and behavior?
	Perceptual fluency	Does making information presented by an online pharmacy easier to process and comprehend (e.g., bullet points *versus* paragraphs) increase purchase intentions and behavior toward that online pharmacy’s medications?
Search	External information search	Is there an inverse relationship between the amount of physician-provided information and online information search? What is the effect of each on online purchase intentions and behavior?
		Does the information provided in direct-to-consumer advertising encourage more extensive information searches and greater online purchase intentions and behavior? How does the effect differ between targeted consumers *versus* the unintended audience also exposed to the message, but with less medical need for the advertised drug?
		To what degree are expatriates likely to seek information about and purchase medicines from their home country versus their country of settlement? How does this impact online medical sales? What methods do expatriates use to locate and purchase medicines online from their home country?
	Involvement/personal relevance	Greater involvement should increase information search as consumers attempt to improve purchase decision quality for more personally relevant decisions. Do the greater health consequences and costs of prescription than OTC medications increase involvement and, hence, information search? How does that affect the amount and type of information that consumers seek when making purchase decisions for a prescription *versus* an OTC medication?
	Intrinsic *versus* extrinsic motivation	Do chronic conditions, due to their ongoing nature, increase enduring involvement and, hence, search for information regarding online medicine purchases compared to acute conditions?
		To what degree do those with chronic diseases prefer to purchase medication online compared to healthy consumers or those with acute conditions? Does the greater frequency of purchasing, the cumulative cost, the difficulty traveling to or navigating a brick-and-mortar pharmacy, or some other factor mediate the relationship between chronic conditions and the greater likelihood of purchasing medications from online sources?
	Search engine optimization Search engine marketing	How can legitimate online pharmacies and brick-and-mortar pharmacies better use search engine optimization and search engine marketing to encourage consumers to switch medication purchases from rogue sites to these legal sellers?
Situational influences	Communication situation	To what degree do consumers intend to or purchase medications via illicit online pharmacies following exposure to direct-to-consumer advertising? Do they first ask their physician for the advertised medication? If so, does a physician’s refusal to prescribe increase or decrease consumers’ likelihood of purchasing the advertised medication online?
	Purchase situation	Does direct-to-consumer advertising of prescription medication impact online purchase intentions or behavior? Is the effect greater among consumer groups with fewer resources (e.g., financial resources or cognitive resources) or among vulnerable populations?
		What is the relationship between online medicine purchases and cross-border sales of medicine? To what degree do consumers travel to purchase medicine or receive medical care *versus* purchase medications online? What accounts for this behavior (e.g., immigration status, living in a borderland region, or medication availability or cost in the country of residence)? What leads consumers to choose online over cross-border medication purchases?
	Usage situation	Are consumers without health insurance more likely to purchase from online pharmacies? If so, how can healthcare providers and public policymakers encourage purchases from legitimate rather than illicit online sources? Comparatively, how effective are different types of interventions (e.g., educational materials, lists of legitimate sources, healthcare provider recommendations)?
	Temporal perspective	How does perceived time pressure impact consumers’ purchase intentions and behavior toward online *versus* brick-and-mortar pharmacies?
	Cognitive tax	How do situations that introduce cognitive taxes, which reduce a consumer’s cognitive capacity remaining for decision-making (e.g., poverty or illness), impact intentions and behavior toward online pharmacies, especially illicit ones that do not require prescriptions?
Familiarity	Prior experience	How does familiarity with purchasing other products online affect online medication purchase intentions and behavior? Do online medication purchase intentions and behavior differ depending on the types of products frequently purchased online?
	Consumer expertise	How does familiarity with a brick-and-mortar chain or independent pharmacy affect consumer purchase intentions and behavior toward an affiliated online pharmacy?
	Objective and subjective knowledge	Does trust, satisfaction with prior purchases, or liking mediate the relationship between online pharmacy familiarity and purchase intentions or behavior?
	Mere exposure	The Enrichment Hypothesis holds that increasing product experience develops consumers’ ability to attend to and retain relevant information about that product. How does familiarity-enhanced attention to and memory for relevant product information affect online medication purchase intentions and behavior? Does greater prior experience alter the distribution of consumers purchasing from legitimate *versus* illicit online pharmacies?
	Enrichment Hypothesis	
Trust	Skepticism	How do consultations with physicians and pharmacists associated with online pharmacies impact consumer trust and skepticism toward online pharmacies or purchase intentions and behavior? Does the modality of the consultation (e.g., email exchange, chat, or video conference) affect these relationships?
		How do shortages and supply chain disruptions affect online purchase intentions and behavior? Do consumers view online access to be sufficiently beneficial to overcome skepticism toward and build trust in online pharmacies, even illicit online sources?
Post-purchase evaluations	Customer satisfaction *versus* dissatisfaction	Are consumers satisfied or dissatisfied with their online pharmacy purchases?
		How satisfied are consumers with the purchase process and the medications received following an online medication purchase?
		Does the degree of satisfaction differ across types of sources for online medication purchases (e.g., legitimate vs. illicit online pharmacy, cost-plus or traditional online pharmacy, chain or independent online pharmacy)?
	Postpurchase dissonance	What impact does post-purchase evaluation have on future purchase intentions and subsequent purchase behavior?
		How common is postpurchase dissonance following an online medication purchase? How does it affect future purchase intentions and behavior toward online pharmacies?

Finally, the prior literature uncovered by this scoping review tends to be atheoretical and exhibits a general lack of definitions for key constructs posited as factors influencing consumer purchase intentions or behavior. To aid pharmacology researchers and investigators from other fields, the research questions in Table 4 are linked to theories and well-defined constructs in the literature from psychology, consumer research, advertising, health communication, and marketing. For example, Fittler et al. (2022b) showed that unlicensed drug distributors use search engine results to redirect site visitors to illegal online pharmacies. Using the constructs from the marketing literature of search engine optimization and search engine marketing, how can legal sellers take action against these rogue sites and use search engine results to promote medication purchases from legitimate online and brick-and-mortar pharmacies? Future research should define the constructs studied and use these theories to establish consistent terminology that will ease comparison across studies to advance understanding of the underlying drivers of consumers’ online medication purchase intentions and behavior.

## 6 Conclusion

This scoping review examined studies of consumers’ online pharmacy purchase intentions and behavior. While demographic factors tended to be the most often measured influences on intentions and behavior, their role was generally inconsistent, with many contradictory results. However, other factors (e.g., convenience, availability, lower prices, favorable evaluations of the purchase environment, and supportive recommendations from others) more consistently enhanced online medication purchase intentions and behavior. These findings of our scoping review should aid verified online pharmacies and brick-and-mortar stores in understanding ways to encourage consumer medication purchases.

Further, our scoping review revealed a general lack of definitions for constructs studied and a paucity of theoretical justification as to why certain factors or individual influences should affect purchase intentions or behavior. This, combined with gaps in the existing literature, led us to develop an extensive agenda for future research to advance our understanding of the drivers of consumers’ intentions and behavior toward online pharmacy purchases. This research agenda should help future studies in this area move beyond descriptive analysis to theory-testing and discovering the underlying explanations of how specific influences affect online medication purchase intentions and behaviors. To accomplish this, these future research directions are based on theories and concepts developed in other areas focused on stimulating intentions or prompting behaviors (e.g., psychology, consumer research, advertising, health communication, and marketing). Investigating research questions based on this agenda should help healthcare providers, public policymakers, and regulators design more effective interventions to promote more positive individual patient and public health outcomes as well as benefit researchers interested in this topic of increasing interest and economic importance.

## Appendix A

Other influences on purchase intentions and behavior identified in prior research.

**Influences on purchase intentions.**

Openness to online medical purchase options (Hu et al., 2021)

Switching costs (Adjie et al., 2023)

Variety-seeking behavior (Adjie et al., 2023)

Fear appeal (Alhabash et al., 2022)

Involvement with online medical products (Lee et al., 2017)

**Influences on purchase behavior.**

*Objectivism* (Rajamma and Pelton, 2009)

Variety seekers (Vera-Martínez, 2023)

*General barriers and website-specific barriers* (Almomani et al., 2023)

Higher number of prescriptions (Brown and Li, 2014)

Have higher Charlson comorbidity index scores (Brown and Li, 2014)

Use of erectile dysfunction drugs (Brown and Li, 2014)

*Use of narcotic medications* (Brown and Li, 2014)

*Satisfaction with pharmacists and physician* (Roblek et al., 2018)

COVID-19 pandemic (Fittler et al., 2022a; Hawdon et al., 2022; Anis and Tan, 2023; Hamdan, 2023)

Pandemic-induced strain (Hawdon et al., 2022)

Note: Italicized text indicates reported findings that are negatively related to purchase intentions or behavior.
